# Temporomandibular joint: from anatomy to internal derangement

**DOI:** 10.1590/0100-3984.2022.0072-en

**Published:** 2023

**Authors:** Lucas Roberto Lelis Botelho de Oliveira, Isabela dos Santos Alves, Ana Patrícia Freitas Vieira, Ula Lindoso Passos, Claudia da Costa Leite, Eloisa Santiago Gebrim

**Affiliations:** 1 Hospital Sírio-Libanês, São Paulo, SP, Brazil; 2 Department of Radiology and Oncology, Faculdade de Medicina da Universidade de São Paulo (FMUSP), São Paulo, SP, Brazil

**Keywords:** Temporomandibular joint, Temporomandibular joint disorders, Temporomandibular joint dysfunction syndrome, Magnetic resonance imaging, Articulação temporomandibular, Transtornos da articulação temporomandibular, Síndrome da disfunção da articulação temporomandibular, Ressonância magnética

## Abstract

The temporomandibular joint can be affected by various conditions, such as joint dysfunction, degenerative changes, inflammatory processes, infections, tumors, and trauma. The aim of this pictorial essay is to help radiologists identify and describe the main findings on magnetic resonance imaging evaluation of the temporomandibular joint, given that the correct diagnosis is essential for the appropriate treatment of patients with temporomandibular joint disorders.

## Abstract (pt)

A articulação temporomandibular pode ser afetada por diversas afecções, como disfunções articulares, alterações degenerativas, doenças inflamatórias ou infecciosas, tumores e trauma. Este ensaio iconográfico visa auxiliar de forma prática o radiologista a identificar e descrever os principais achados nos exames de ressonância magnética da articulação temporomandibular, tendo em vista que o diagnóstico correto das alterações mais comuns é essencial para o tratamento adequado desses pacientes.

## INTRODUCTION

The temporomandibular joint (TMJ) is a complex synovial joint that can be affected by various conditions, such as joint dysfunction, degenerative changes, inflammatory processes, infections, tumors, and trauma. The aim of this pictorial essay was to review the anatomy of the TMJ, as well as derangement and degenerative changes within the joint, discussing the main findings on magnetic resonance imaging (MRI).

## NORMAL ANATOMY OF THE TMJ

The bony portion of the TMJ is composed of a temporal component and a mandibular component (the mandibular condyle). Each component is surrounded by a synovial capsule. The joint cavity is composed of the squamous portion of the temporal bone, whose anterior border is the articular eminence. The mandibular condyle projects into the joint cavity and typically has an oval or rounded morphology^(1,2)^.

**Figure 1:**
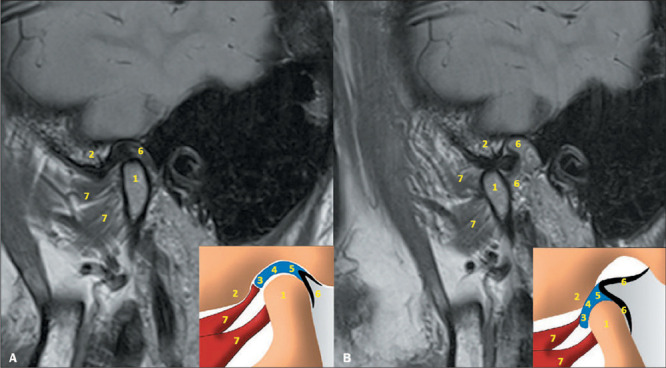
TMJ anatomy. Sagittal PD-weighted images and schematic drawings demonstrating the anatomy of the TMJ in closed-mouth and open-mouth positions (**A** and **B**, respectively). 1, mandibular condyle; 2, articular eminence of the temporal bone; 3, articular disc (anterior band); 4, articular disc (intermediate band); 5, articular disc (posterior band); 6, bilaminar zone; 7, superior and inferior bellies of the lateral pterygoid muscle.

The TMJ disc is a biconcave, fibrocartilaginous structure that interposes between the mandibular condyle and the articular surface of the temporal bone. This interposition of the disc between the two bony portions of the joint has the important function of preventing joint damage. In addition, the disc facilitates the sliding of the mandibular condyle in relation to the temporal bone during the opening and closing of the mouth. Because of the biconcave shape of the disc, its peripheral zones (the anterior and posterior bands) are thicker than its central portion (the intermediate zone). The disc is attached to the inner surface of the TMJ capsule, creating two joint cavities—an upper cavity and a lower cavity. Under normal conditions (Figure 1), there is no communication between those two cavities^(3,4,5)^.

The TMJ disc is stabilized posteriorly by the bilaminar zone, which inserts into the posterior portion of the posterior band of the disc. The bilaminar zone, also known as the retrodiscal tissue, has two layers of fibers, an upper layer (the temporal lamina), which inserts into the anterior contour of the external auditory canal, and a lower layer, which inserts into the posterior contour of the mandibular condyle. The layers are separated by elastic tissue containing vessels and nerves. The bilaminar zone has the function of preventing slippage or excessive rotation of the disc during mouth opening. There is also a small lamina anterior to the articular eminence and mandibular head^(3,4,5)^.

The lateral pterygoid muscle has two bellies. The superior belly inserts on the anteromedial surface of the neck of the mandible, in the pterygoid fovea region, and, in some patients, part of the superior belly inserts on the surface of the TMJ capsule. The inferior belly also inserts into the pterygoid fovea^(3,4,5)^.

## IMAGING METHODS

### Radiography

Conventional X-ray of the TMJ, with oblique transcranial projection performed in profile in the open- and closed-mouth positions (Schuller’s view), provides an overview of the bones that make up the joint and joint space. However, it does not allow an assessment of the cartilage or adjacent soft parts, in addition to being impaired by the overlap with other cranial bone structures^(6)^.

### Computed tomography

Computed tomography presents excellent image resolution, mainly for the evaluation of bone structures, making it the method of choice when evaluating fractures involving the TMJ or the involvement of the cortical bone by tumors or inflammatory processes. Computed tomography also allows three-dimensional reconstructions, which are very useful in fracture assessment and surgical planning^(6,7)^.

## MRI

For the investigation of TMJ disorders, the modality of choice is MRI. Images ≤ 3 mm in thickness should be acquired in proton density (PD)-weighted, T1-weighted, and T2-weighted sequences. As detailed in Table 1, PD-weighted sequences should be obtained in the coronal and sagittal planes in the closed-mouth position, as well as in the sagittal plane in the open-mouth position; the T1-weighted sequences should be obtained in the sagittal plane in the closed-mouth position; and the T2-weighted sequences should be obtained in the coronal and sagittal planes in the closed-mouth position. It is important that the images are acquired in the sagittal and coronal planes oblique to the TMJ. Paramagnetic contrast should be used when an inflammatory process or tumor is suspected. Dynamic assessments can also be used^(6,7,8)^.

**Table 1:** Suggested MRI protocol for TMJ assessment at our facility.

Plane	Weighting	Slice thickness (mm)	Position
Axial	T2	3.0	Closed-mouth
Coronal	DP	2.5	
Oblique sagittal	T1	2.5	
	T2	2.5	
	DP	2.5	
	DP	2.5	Open-mouth

## NORMAL APPEARANCE OF THE TMJ

The TMJ disc usually has a biconcave shape and homogeneously low or intermediate signal intensity on T1- and T2-weighted images. In some cases, there can be an area of discrete high signal intensity in the central portion of the disc on T2- and PD-weighted images. The posterior insertion of the disc has a signal that is hyperintense to the muscle on PD- and T1-weighted images, due to the presence of fatty tissue, whereas the bilaminar zone shows a signal isointense to muscle in all sequences. The posterior band and the bilaminar zone are best evaluated in the open-mouth position^(1,7)^.

**Figure 2:**
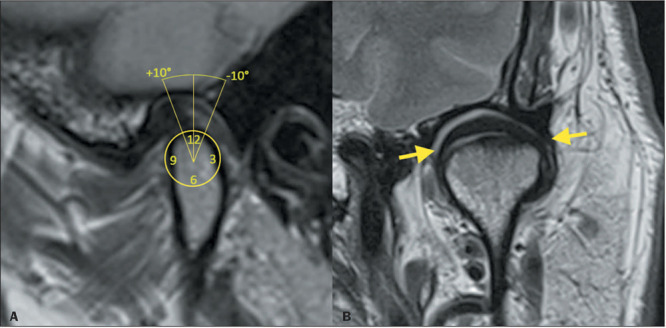
Normal TMJ disc position. Sagittal and coronal PD-weighted images obtained in the closed-mouth position (**A** and **B**, respectively). Note that the junction of the posterior band of the articular disc and the bilaminar zone is near the 12 o’clock position or situated within 10° of that position (**A**). In the coronal plane (**B**), the articular disc (arrows) should not (and does not, in this normal image) extend beyond the medial or lateral edge of the condyle.

**Figure 3:**
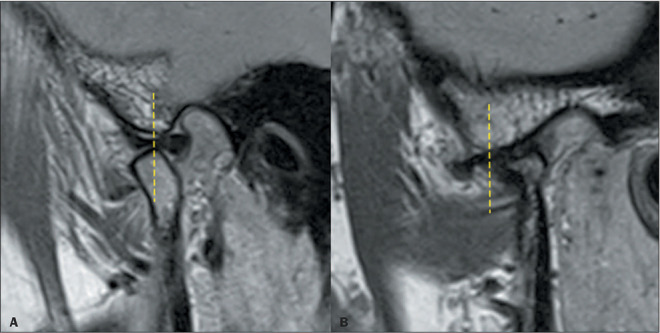
Condylar excursion. Sagittal PD-weighted images in the open-mouth position, showing excursion of the condyle. The dashed lines indicate the position of the articular eminence of the temporal bone. **A:** Appropriate excursion. Note that the mandibular condyle articulates with the temporal eminence in the open-mouth position. **B:** Decreased excursion. The condyle does not reach the plane of the temporal eminence in the open-mouth position. Note the degenerative changes, characterized by flattening of the condyle with irregularities of the articular surface, subchondral sclerosis, and an anterior osteophyte. In cases of increased excursion, the condyle passes beyond the temporal eminence in the open-mouth position.

In the closed-mouth position, the junction between the posterior band of the disc and the bilaminar zone is located near the 11 or 12 o’clock position (Figure 2). When the mouth opens, the condyle makes an anterior translation until it articulates with the articular eminence (Figure 3). In the coronal plane, the medial and lateral edges of the disc are aligned with the edges of the condyle, not projecting laterally or medially^(1,3,4,5)^.

## TEMPOROMANDIBULAR DISORDER

Temporomandibular disorder is a common condition that affects up to 28% of the general population; the term encompasses a wide range of conditions involving the TMJ^(9)^.

## INTERNAL DERANGEMENT

The main cause of TMJ dysfunction is the internal derangement of the joint, defined as an abnormal relationship between the articular disc and the mandibular condyle that interferes with the normal biomechanics of the TMJ and can manifest clinically as joint pain, jaw deviation while opening the mouth, or sounds (e.g., clicks) emanating from the joint^(7)^.

### Disc displacement

**Figure 4:**
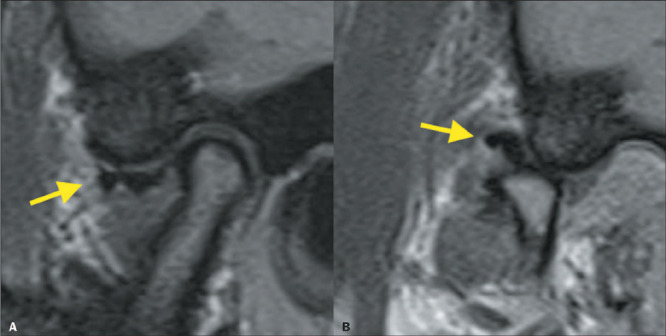
Anterior disc displacement without recapture. Sagittal PD-weighted image in the closed-mouth position (**A**), showing the disc anterior to the 11 o’clock position. In the open-mouth position (**B**), there is no disc recapture. The disc position is indicated by the arrows.

**Figure 5:**
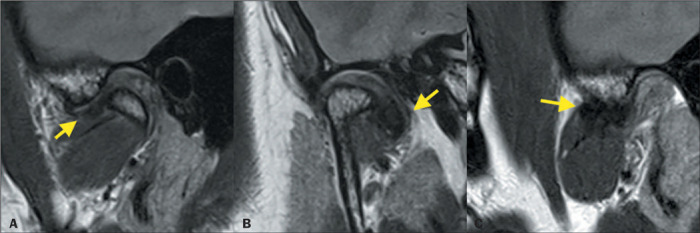
Anteromedial disc displacement without recapture. Sagittal PD-weighted image in the closed-mouth position (**A**), showing anterior disc displacement. Coronal image (**B**) showing the disc in a medial location. In the PD-weighted sagittal acquisition in the open-mouth position (**C**), the condyle is not observed in the plane in which the disc is visualized, demonstrating that the disc remained in the medial location.

**Figure 6:**
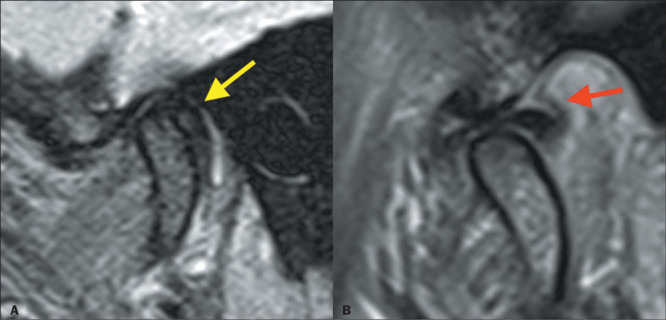
Posterior disc displacement with recapture. Sagittal PD-weighted images in the closed-mouth and open-mouth positions (**A** and **B**, respectively). Posterior displacement of the articular disc (yellow arrow) is accompanied by bilaminar thickening (red arrow) and joint effusion. Note the recapture of the articular disc (in **B**).

The most common cause of internal derangement of the TMJ is disc displacement. The position of the TMJ disc is evaluated according to its relationship to the condyle. Disc displacement can thus be classified as anterior, posterior, medial, or lateral. There can also be multidirectional displacement, which is classified as anterolateral or anteromedial. Although the most common type of displacement is anterior (Figure 4), anteromedial or anterolateral displacement is often seen (Figure 5). Posterior displacement is rare (Figure 6). Disc displacement can also be partial or total. It is categorized as partial when the posterior band is in a normal position in at least one sagittal plane image and as total when the posterior band is in an abnormal position in all sagittal plane images. Total displacement is typically seen in symptomatic patients, whereas partial displacement is more common in asymptomatic patients^(8,9,10)^.

Anterior displacement of the TMJ disc is defined as a condition in which the junction of the posterior band and the bilaminar zone is more anterior than the 11 o’clock position or in which the angle between the posterior band of the disc and a vertical line running through the condylar head is greater than 10°. However, to make a definitive diagnosis based on that criterion, it is necessary to consider the clinical correlation, given that small anterior disc displacements can be seen in up to 33% of asymptomatic individuals. Other authors have proposed that the intermediate zone be the reference point for assessing disc position, which is considered normal when the disc is interposed between the anterior eminence of the condyle and the posterior aspect of the articular eminence of the temporal bone. Medial and lateral displacements are more easily characterized in the coronal plane, in which the disc should not extend beyond the lateral or medial edge of the condyle. This is also evaluated in sagittal acquisitions, in which the disc should not appear medial or lateral to the condyle^(9,11)^.

An investigation of disc displacement should include the evaluation of recapture, which occurs when the disc returns to its normal position after mouth opening. Anterior displacement with recapture can be seen in asymptomatic individuals who do not require treatment. Anterior displacement without recapture is commonly associated with joint disc deformity, joint effusion, or disc perforation, as well as with bone changes such as edema, erosion, or necrosis of the condyle and temporal eminence^(11)^.

### Disc morphology

**Figure 7:**
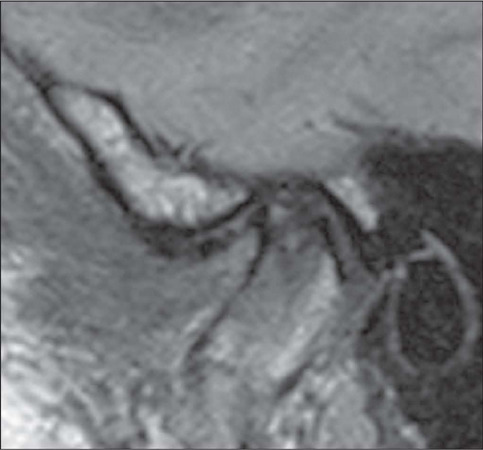
Degenerative disc changes. Sagittal PD-weighted image in the closed-mouth position, showing anterior disc displacement and morphological changes, with tapering and irregularities of the articular disc.

In the early stages of TMJ dysfunction, the disc retains its usual shape and signal characteristics. As the disease progresses, the disc undergoes degenerative changes, with thickening of the posterior band and thinning of the anterior band, and can acquire a biconvex or rounded shape (Figures 6 and 7).

**Figure 8:**
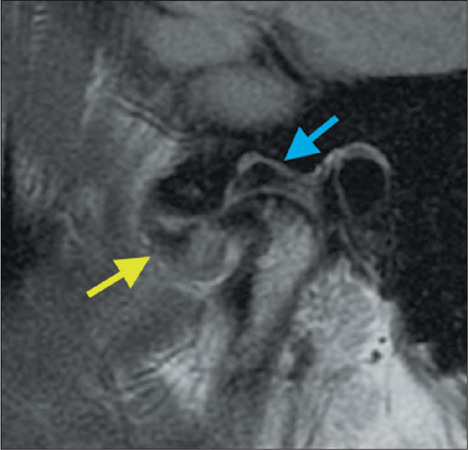
Disc perforation. Sagittal PD-weighted image showing discontinuity of the articular disc, with separation of the anterior band (yellow arrow) and posterior band (blue arrow). Note also the subcortical sclerosis with an anterior marginal osteophyte in the mandibular condyle.

**Figure 9:**
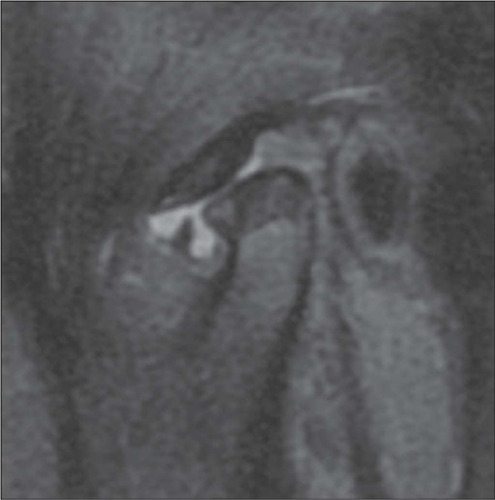
Joint effusion. Sagittal T2-weighted image with fat saturation, showing joint effusion. Note the communication between the upper and lower joint cavities, indicating disc perforation.

In the advanced stages of TMJ dysfunction, disc perforation occurs in 5–15% of cases, being most common in those of disc displacement without recapture. There is also a relationship between the type of disc displacement and the location of disc perforation^(8,12)^: anterolateral displacement is associated with perforation of the medial aspect of the disc, whereas anteromedial displacement is associated with perforation of the lateral aspect (Figure 8). Disc perforation is more easily identified in cases in which it is accompanied by joint effusion (Figure 9) and is confirmed by detecting communication between the superior and inferior joint cavities^(9,11)^.

### Retrodiscal tissue

**Figure 10:**
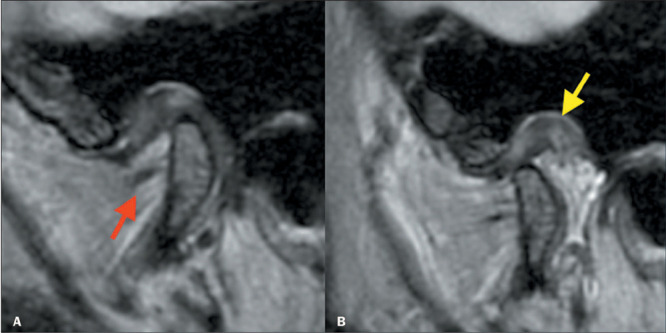
Disc adhesion. PD-weighted sagittal images in the closed-mouth and open-mouth positions (**A** and **B**, respectively), showing alteration of the signal in the posterior band and intermediate zone of the articular disc, together with thickening of the retrodiscal tissue (yellow arrow). Note that the disc remains in the same location in the closed-mouth and open-mouth positions, which is characteristic of disc adhesion (stuck disc). Note also the thickening at the insertion of the lateral pterygoid muscle belly (red arrow), forming the double-disc sign.

In some patients with anterior disc displacement, adaptive changes may occur. Such changes include thickening and hyalinization of the upper and lower disc layers that constitute the retrodiscal tissue (Figure 6), resulting in loss of the usual high signal intensity of the disc on T1-weighted sequences, on which it presents as a pseudodisc. On T2-weighted images, the retrodiscal tissue can also show a hyperintense signal (Figure 10), which is related to local hypervascularization and joint pain^(9)^.

### Disk adhesions

As the internal derangement progresses, adhesions can develop. Those adhesions can keep the TMJ disc in a fixed (mouth-closed or mouth-open) position. This condition, known as a stuck disc, significantly limits condylar translation^(9)^, as illustrated in Figure 10.

### Double-disc sign

Electroneuromyography studies have identified hyperactivity of the inferior belly of the lateral pterygoid muscle in patients with internal derangement of the TMJ^(9)^. In such patients, there can be thickening at the insertion of the inferior belly of the lateral pterygoid, forming the double-disc sign (Figure 10).

### Joint effusion

**Figure 11:**
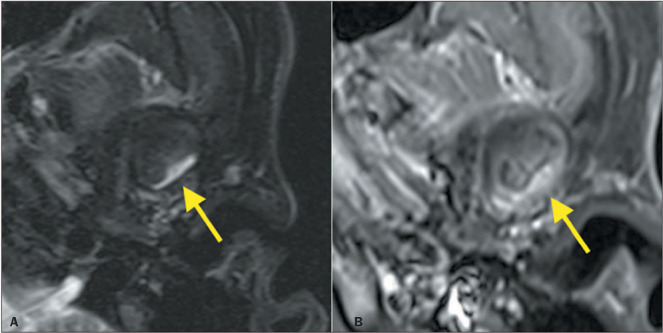
Synovitis. Axial T2-weighted image with fat saturation (**A**) and contrast-enhanced T1-weighted image (**B**), showing synovial thickening with enhancement consistent with synovitis, which should not be confused with joint effusion.

A small amount of fluid can be seen in normal joints. Small joint effusions are commonly seen surrounding the anterior band of the articular disc (Figure 9). Moderate or severe effusion is associated with pain and disc displacement, typically being a precursor of bone degeneration^(9,11)^. If osteoarthritis is suspected, contrast-enhanced T1-weighted sequences help differentiate between synovitis and joint effusion. On T2-weighted sequences, a thickened synovium typically shows a hyperintense signal and contrast enhancement (Figure 11); synovitis should not be confused with joint effusion, in which there will be no enhancement^(13)^.

## DEGENERATIVE CHANGES IN THE TMJ

In general, degenerative changes represent an advanced stage of TMJ dysfunction after a long process of disc displacement without recapture, which results in deterioration and abrasion of the articular cartilage, with thickening and remodeling of the underlying bone, and, in some cases, the presence of synovitis signs, bone edema, reduced joint space, irregular condylar margins, osteophytes, and bone sclerosis. Paradoxically, symptoms tend to decrease with age, becoming self-limiting and remitting^(11)^.

## CONCLUSION

Internal derangement, together with its possible complications and sequelae, is the pathological condition most commonly seen in the TMJ (Table 2). Symptoms involving pain or sounds (e.g., clicks) are nonspecific. Therefore, in order to perform an accurate assessment of patients with such symptoms, it is important to have knowledge of TMJ anatomy and biomechanics, as well as of the normal and pathological imaging findings.

**Table 2:** Main imaging findings of TMJ internal derangements.

**Direct signs**
● Abnormal disc position:
– Anterior displacement (the most common type): the junction of the posterior band of the disc and the bilaminar zone is more than 10° from the vertical line running through the condylar head
– Posterior displacement (a rare type): the posterior band is more than −10° from the vertical line running through the condylar head costly
– Medial or lateral displacement: the disc extends beyond the edges of the condyle in the coronal plane
● Disc abnormalities: irregularities, rounding, flattening, or perforation
● Abnormalities of movement:
– Reduced excursion
– Displacement: presence or absence of recapture
– Stuck disc: disc remains fixed during mouth opening and closing
Sinus tract, simple fistula, inflammatory mass, free perforation, abscess
● Condylar osteoarthritis: irregularities, erosions, sclerosis, and osteophytosis
**Indirect signs**
● Joint effusion
● Thickening at the insertion of the inferior belly of the lateral pterygoid muscle: double-disc sign
● Thickening of the bilaminar zone: pseudo-disc

## References

[1] Alomar X, Medrano J, Cabratosa J (2007). Anatomy of the temporomandibular joint. Semin Ultrasound CT MR.

[2] Sommer OJ, Aigner F, Rudisch A (2003). Cross-sectional and functional imaging of the temporomandibular joint:
radiology, pathology, and basic biomechanics of the jaw. Radiographics.

[3] Tamimi D, Jalali E, Hatcher D (2018). Temporomandibular joint imaging. Radiol Clin North Am.

[4] Salamon NM, Casselman JW (2020). Temporomandibular joint disorders: a pictorial review. Semin Musculoskelet Radiol.

[5] Tamimi D, Kocasarac HD, Mardini S (2019). Imaging of the temporomandibular joint. Semin Roentgenol.

[6] Bag AK, Gaddikeri S, Singhal A (2014). Imaging of the temporomandibular joint: an update. World J Radiol.

[7] Petscavage-Thomas JM, Walker EA (2014). Unlocking the jaw: advanced imaging of the temporomandibular
joint. AJR Am J Roentgenol.

[8] Rao VM, Bacelar MT (2002). MR imaging of the temporomandibular joint. Magn Reson Imaging Clin N Am.

[9] Tomas X, Pomes J, Berenguer J (2006). MR imaging of temporomandibular joint dysfunction: a pictorial
review. Radiographics.

[10] Morales H, Cornelius R (2016). Imaging approach to temporomandibular joint disorders. Clin Neuroradiol.

[11] Aiken A, Bouloux G, Hudgins P (2012). MR imaging of the temporomandibular joint. Magn Reson Imaging Clin N Am.

[12] Liu XM, Zhang SY, Yang C (2010). Correlation between disc displacements and locations of disc perforation in
the temporomandibular joint. Dentomaxillofac Radiol.

[13] Navallas M, Inarejos EJ, Iglesias E (2017). MR imaging of the temporomandibular joint in juvenile idiopathic arthritis:
technique and findings. Radiographics.

